# Genomic analysis of differentiation and demography of the formerly conspecific agile (*Dipodomys agilis*) and Dulzura (*D. simulans*) kangaroo rats

**DOI:** 10.1038/s41437-025-00789-3

**Published:** 2025-08-25

**Authors:** Yuwei Cui, Leonard Nunney

**Affiliations:** Department of Evolution, Ecology, and Organismal Biology, University of California Riverside, Riverside, CA USA

**Keywords:** Evolutionary genetics, Speciation

## Abstract

Karyotype variation within Pacific kangaroo rat *Dipodomys agilis* motivated its division in 1997 into the agile kangaroo rat (AKR, *D. agilis*, 2N = 62) in the north of its range in California, and Dulzura kangaroo rat (DKR, *D. simulans*, 2N = 60) to the south, with a suspected sympatric zone south of the San Gabriel and San Bernardino Mountains. This division was supported by our whole genome sequencing that sampled a ~120 km transect from north of the mountains to SW Riverside County. The taxa showed marked genetic differentiation, with no evidence of hybridization or sympatry. AKR was found at the southern edge of the mountains, precluding the mountain barrier driving isolation, suggesting ecological separation linked to habitat differences between the mountains and the arid area to the south. Adding four additional *Dipodomys* species, we estimated genetic divergence times in the genus back to ∼3.5 mya. AKR and DKR diverged from *D. stephensi* ∼1.7 mya, and from each other ∼0.5 mya, when their joint effective population size (*N*_*e*_) was ~100,000. After separation, DKR’s *N*_*e*_ declined to ~20,000, while AKR’s was little changed. More recently their *N*_*e*_ converged at ~50,000. Runs of homozygosity were longer in AKR, indicating a smaller neighborhood size, which may have promoted the karyotype change; however, nucleotide diversity was higher in AKR, but both had levels typical for rodents, indicating neither experienced recent bottlenecks. These patterns provide a baseline for any future conservation efforts. More generally, this study shows how a detailed genomic study can resolve taxonomic and demographic questions among morphologically indistinguishable taxa.

## Introduction

Species are mainly defined by their geographical distribution and their morphological traits but other features ranging from behavior to karyotype may also be used. However, species delineation can be complicated by historical gene flow and current hybridization between taxa. In the past, gene introgression and hybridization were generally difficult to detect; however, with the development of molecular and genomic tools, cases have been found in a wide variety of species (Baack and Rieseberg 2007), including birds (Burri et al. 2015; Han et al. 2017), insects (Pardo-Diaz et al. 2012), and plants (Suarez-Gonzalez et al. 2018; Wong et al. 2022). Even in such a well-studied group as mammals, recent genomic studies suggest that there are more hybrid zones, gene introgression, and cryptic isolation between taxa than previously acknowledged (Beysard et al. 2012; Shurtliff 2013; Fontsere et al. 2019; Kubiak et al. 2020; Ge et al. 2022; Herrera et al. 2020), emphasizing the importance of genomic data in confirming ambiguous species delineations.

One factor that can minimize gene flow and so promote reproductive isolation and speciation is the development of karyotype differences within a species (Castiglia 2014). Karyotype differences can inhibit hybridization if there is a fitness loss in chromosomal heterozygotes due to meiotic incompatibilities. For example, in the western house mouse (*Mus musculus domesticus*), the reproductive isolation between the Poschiavo (2N = 26) and the Upper Valtellina (2N = 24) chromosomal races is likely a result of reduced fertility in hybrids (Hauffe and Searle 1998; Giménez et al. 2013).

However, such fitness loss is not inevitable. Karyotype polymorphism resulting from a Robertsonian fusion (the joining of two acrocentric chromosomes at their centromeric region) is widespread in mammals suggesting such chromosomal heterozygotes suffer little or no fitness loss (Dobigny et al. 2017). Examples indicating minimal or no fitness loss in chromosomal heterozygotes within populations include shrews of the *Sorex araneus* group (Horn et al. 2012), the southern short-tailed shrew (*Blarina carolinensis*) (Qumsiyeh et al. 1997) and the Brazilian marsh rat *Holochilus brasiliensis* (Nachman 1992). Over the longer term, Jensen et al. (2023) showed that within the guenon group of primates there has been frequent gene flow among lineages with different chromosome numbers, a pattern also seen in rock wallabies (Potter et al. 2015). Such observations indicate that karyotype differences may not always be a good indication of reproductive isolation or species level status, again emphasizing the importance of genomic data in determining genetic isolation and species delineation.

The complexity of defining taxonomic status in the absence of genomic data is apparent in the kangaroo rats (*Dipodomys* spp.). First, closely related kangaroo rat species show very little morphological difference so delimiting species based solely on morphology is often very difficult. Second, intraspecific karyotype polymorphism has been found in several species, including *D. panamintinus*, *D. spectabilis*, and *D. microps* (Stock 1974; Csuti 1979), so delineating species based on karyotype needs a strong justification. This complexity is apparent in the case of the taxa previously grouped together and classified as the Pacific kangaroo rat (PacKR, *Dipodomys agilis*).

Prior to 1997, PacKR was considered a single species divided into a number of geographically separated subspecies with a range extending from Tulare County in California to Magdalena Plain of southern Baja California (Best 1978; 1983). Chromosomal analyses showed karyotype differences between the northern subspecies (subspp. *agilis* and *perplexus*) with 2N = 62 (Csuti 1971) and the southern subspecies (subspp *simulans* and *plectilis*) with 2N = 60 (Stock 1974). This karyotype difference was the primary basis for redefining PacKR as two species, *D. agilis*, now given the common name of the agile kangaroo rat (AKR), and *D. simulans*, the Dulzura kangaroo rat (DKR) (Sullivan and Best 1997). Sullivan and Best (1997) supported their karyotype-based reclassification of PacKR with a morphological analysis that showed animals from southern populations in Mexico were significantly larger than those from sites about 400 km further north (north of Los Angeles). A similar north-south morphological trend had been previously noted (Best 1983; Best et al. 1986), and grouping populations by karyotype was found to explain about 20% of the morphological variance (Best et al. 1986). Allozyme gene frequency data also showed that individuals from northern populations (2N = 62) clustered together genetically (Best et al. 1986), although they concluded that these minor differences were probably the result of a recent geographical separation of the two chromosomal forms.

Sullivan and Best (1997) also examined animals of unknown karyotype and classified them by morphology. Their results suggested that the two species had a broad zone of sympatry from the southern foothills of the San Gabriel and San Bernardino Mountains south to the Santa Ana Mountains. This raised the possibility of ongoing or historical hybridization and genetic introgression, possibly at a level inconsistent with the species designations.

The subdivision of the Pacific kangaroo rat into the two species, AKR and DKR, has been generally accepted, although there remains a lack of compelling genetic support for the split. Apart from the inconclusive allozyme data, the only other genetic data were part of a phylogenetic study of heteromyids based on two mitochondrial genes that showed divergence of the two taxa; however, the sequences were from individuals from two sample sites >400 km apart (Alexander and Riddle 2005), so the divergence could be explained by isolation-by-distance. Thus, while the karyotype difference could indicate a species-level difference between the taxa, the supporting data remain relatively weak.

To evaluate the degree of genetic isolation between the taxa, we used genomic data from individuals sampled along a north-south transect from the northern edge of the San Gabriel Mountains to the lower elevation lands to the south in the western Riverside County.

Sullivan and Best (1997) hypothesized that AKR are distributed at higher elevations (>800 m) in the San Gabriel, San Bernardino, and coastal Santa Monica mountains while DKR occurs at lower elevations (<800 m) south from the foothills of those mountains, suggesting that elevation may be a major environmental factor influencing the distribution of these two species in the area of potential sympatry. To test this hypothesis of ecological separation, we examined (a) if there was evidence of sympatry, and if so were the taxa locally separated by elevation, and (b) in the absence of sympatry, could the geologically recent rise of the San Gabriel and San Bernardino mountains have created a physical barrier driving vicariant speciation, as appears to be the case for the split between Stephens’ kangaroo rat (SKR, *D. stephensi*) and the Panamint kangaroo rat (PKR, *D. panamintinus*) (Metcalf et al. 2001), or do ecological factors associated with elevation provide a more plausible explanation.

To determine whether AKR and DKR are distinct species, we estimated the genome-wide differentiation, the level of genetic admixture, and the timing of any divergence between them. We also examined whether the divergence of the two taxa has been associated with any notable bottlenecks or other changes in their historical effective population sizes (*N*_*e*_). Determining their past patterns of population size is relevant to understanding their levels of genetic variation and to guide any future conservation strategies, given that, while neither species is currently considered to be under threat, both taxa live in a region experiencing a rapid loss of natural habitat.

## Methods

### Field sampling and sample preservation

**Fig. 1:**
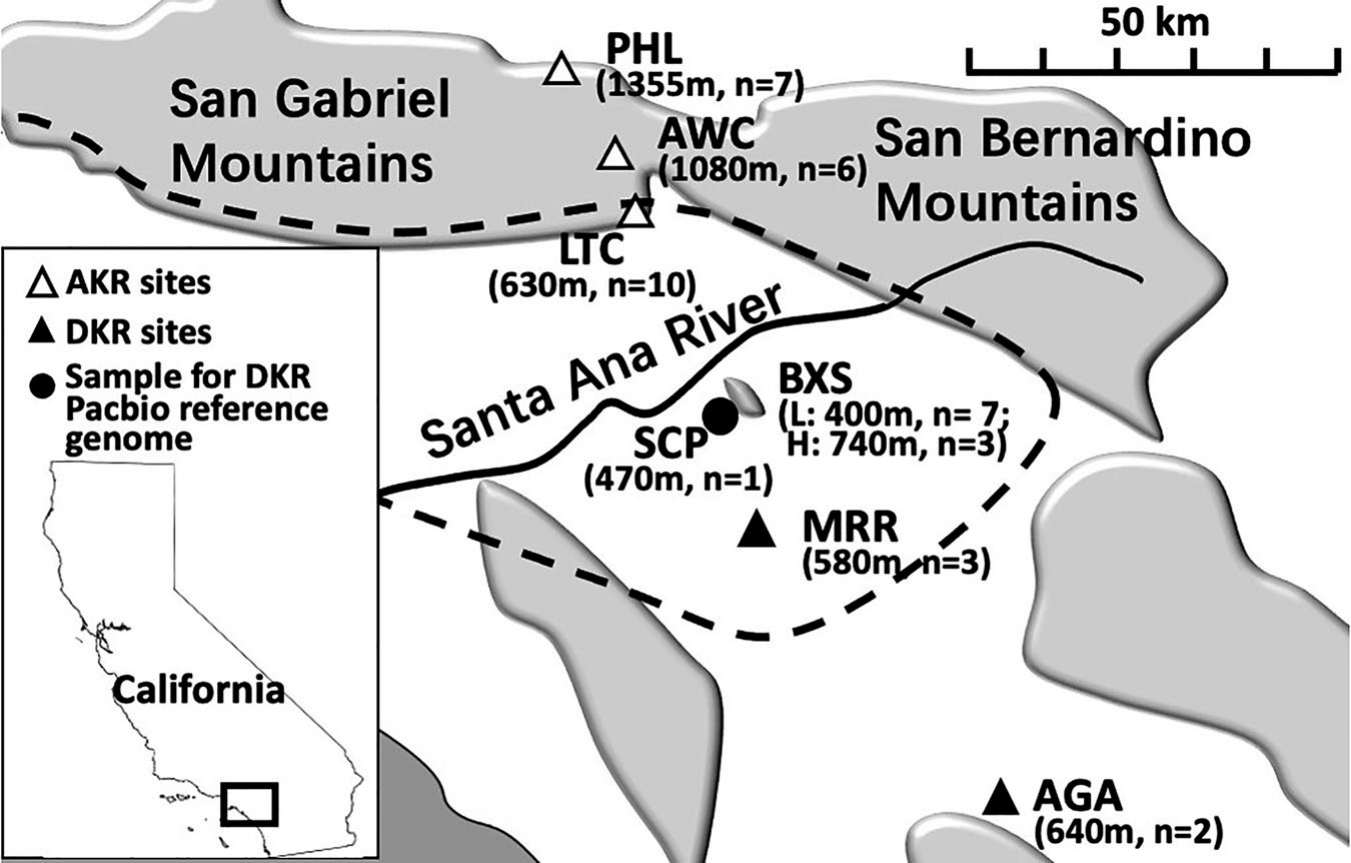
Sampling sites and the location of the salvaged animal. The elevations and samples sizes (*n*) are shown in parentheses below the sample site abbreviations. For full names of sample sites, see Methods. The shading defines major mountain ranges, the black line defines the Santa Ana River, and the dashed lines encloses the possible zone of AKR/DKR sympatry, as defined by Sullivan and Best (1997).

Six sites from along a ~120 km transect crossing the San Gabriel mountains and the foothills to the south were selected for sampling (Fig. 1). Based on the conclusions of Sullivan and Best (1997), two sites within the San Gabriel mountains were chosen that were expected to be exclusively AKR (Phelan, PHL, Applewhite Camp, AWC, both >1000 m elevation), with one site exclusively DKR in SW Riverside County (Aguanga, AGA, at 640 m elevation). Between them, four sites were within the area of sympatry proposed by Sullivan and Best (1997): Lytle Creek, LTC at the southern edge of the mountains, at 630 m; two sites within the Box Springs Mountains, Box Springs high (BXSH, 740 m elevation) and Box Springs low (BXSL, 400 m elevation); and the UC Motte Rimrock Reserve, MRR, 580 m. The two Box Springs Mountains sites were sampled because Sullivan and Best (1997) had proposed that the two species might be separated by elevation.

Sherman live traps were used to capture the rodents, and ~10 mg ear slices were cut from both ears of the targeted kangaroo rats. The samples were stored on dry ice in the field and then put into −80 °C freezer. Trapping and sampling were approved under California Dept. of Fish and Wildlife Scientific Collecting Permit SC-11898 and UC Riverside Institutional Animal Care and Use Committee (IACUC) Animal Use Protocol 20140001 and 20180008.

### Reference genome sequencing and assembly

A dead animal was salvaged at the Sycamore Canyon Park (SCP) and, given the location, identified as a probable DKR. It was used to assemble a reference genome, with its identity to be confirmed by genomic comparison with the other sampled animals. The whole animal was stored at −80 °C. We defrosted and dissected the animal and sent the whole heart (~250 mg) to UC Davis genome center, where the high molecular weight DNA (HMW DNA) was extracted, and quality check was performed prior to Pacific BioSciences (PacBio) sequencing (Eid et al. 2009). The sequencing was performed using 5 single-molecule real-time (SMRT) cells on Sequel II sequencer to generate High-Fidelity (HiFi) Long Reads. The HiFi reads of PacBio were assembled using hifiasm (Cheng et al. 2021) to obtain a de novo genome of this non-model organism, which was used as a reference for our population genomics studies. The basic statistics of this assembly were obtained using a python script (https://github.com/PacificBiosciences/pb-assembly/blob/master/scripts/get_asm_stats.py). To get the sequencing depth, the PacBio reads were aligned to the assembled contigs using minimap2 v2.24 (Li 2018), and samtools v1.14 (Li et al. 2009) was used to estimate the coverage. The completeness of the assembly was checked by BUSCO (Benchmarking Universal Single-Copy Orthologs) (Simão et al. 2015) using the mammalia_odb10 lineage dataset, which looked for 9226 conserved single-copy orthologs found in mammals.

### Genome re-sequencing, alignment and SNP calling

Genomic DNA was extracted from one ear slice of each sampled animal using Qiagen DNeasy Blood & Tissue Kits (except for one DKR sample - see below). The whole genome re-sequencing was performed at the UC Davis genome center, where 37 samples were pooled with 23 samples for other projects (60 samples in total) and barcoded for library preparation and sequenced on 2 lanes of an Illumina NovaSeq S4 sequencer for 150 bp paired end (PE) reads, aiming at a coverage at 4–8× per sample.

One AKR and one DKR individual were subject to higher reading depth sequencing and were used when the genetic analysis required higher quality mapping to the reference genome. The AKR sample used was from one of the individuals captured at AWC. Higher coverage was achieved by preparing 9 libraries instead of the one library prepared for each of the other samples. The higher quality DKR sequence was from an individual captured at AGA and derived from 10X linked-reads genomic sequencing (https://www.10xgenomics.com/) by HudsonAlpha Institute for Biotechnology (https://hudsonalpha.org/). This method uses unique marking of all short reads from each specific long DNA molecule (Marks et al. 2019). The 10X sequencing of an AGA DKR was done prior to our receiving the salvaged DKR from SCP used for PacBio. We did not re-sequence the SCP sample because we already had one DKR sample with high quality Illumina data and SCP was not one of our sample sites (see Fig. 1).

The reads were trimmed by TrimGalore (https://github.com/FelixKrueger/TrimGalore) with the pass quality set as default (Phred score ≥20) and adapters automatically detected. The forward and reverse reads were merged using PEAR v0.9.6 (Paired-end read merger; Zhang et al. 2014) if the paired reads overlap each other, and the merged and unmerged reads were aligned to our PacBio assembly using Burrows-Wheeler Aligner v 0.7.17 (BWA; Li and Durbin 2009). The alignment was processed using samtools v1.16 (Li et al. 2009), and SNP calling was performed by bcftools mpileup function (Danecek et al. 2021). In addition, the consensus sequence of AKR was obtained by aligning the reads of the high coverage AKR sample to our reference assembly using samtools and converted into fasta file using Seqtk (Li 2013).

### Population structure

To detect population structure and potential admixture, we used Admixture v1.3.0 (Alexander et al. 2009) to estimate the global ancestry of the genomes of the samples, varying the number of clusters (K) tested from 1 to 10. First, vcftools v 0.1.16 (Danecek et al. 2011) was used to filter the variant sites, for which the minor allele frequency (MAF) was set as 0.05 and the maximum missing data as 20%. To run Admixture with a large number of contigs, admixturePipeline v3.0 (Mussmann et al. 2020, https://github.com/stevemussmann/admixturePipeline) was used to pre-process the variants. CLUMPAK (Cluster Markov Packager Across K, Kopelman et al. 2015) was used to process the output of Admixture and identify the optimal clustering. The best K was estimated using an ad hoc statistic, Delta K, which measures how much log probability changes between successive Ks (Evanno et al. 2005). Additionally, we carried out a principal component analysis (PCA) using Plink v1.90b6.25 (Purcell et al. 2007), which transforms the genetic variation into principal components to identify population clusters.

To further quantify the genetic variation, we used variable sites to estimate genome-wide Fst (Wright 1949) using vcftools v 0.1.16-18, which calculates the Weir and Cockerham (1984) modification of Fst. To control for the genotyping errors due to low sequencing depth (resulting in some heterozygotes being misclassified as homozygotes), high sequencing depth due to misalignment of repetitive DNA (resulting in overestimate of variability), sequencing errors, and the effect of missing data, we filtered the alignment to keep sites with an individual reading depth between 5 × to 16 ×, overall missing data <20%, and a minor allele count >1, following the approach outlined in Lou et al. (2021). The two high coverage samples (from AGA and AWC) were excluded to maintain consistency of the reading depth filter.

To compare Fst values within and between the taxa, we used a simple one-tailed permutation test, recognizing the non-independence of the different Fst values involving any specific site. For each of the seven sites, the average Fst within and between taxa was calculated and compared.

### Genomic variation within populations

Pixy v1.2.7 (Korunes and Samuk 2021) was used to estimate the genome wide nucleotide diversity (π) for the low coverage samples based on sites filtered by the same criteria used for Fst estimates, with the only difference being that the invariant sites were also used, filtered on reading depth. A Welch’s *t* test was used to compare π between the two taxa.

Runs of homozygosity (ROHs) were estimated using bcftools. Since ROHs define a short-term measure determined by the local mating patterns, we tested whether the population structure was different in the two taxa and in different populations by comparing the individual mean ROH lengths using a nested ANOVA test with populations nested within species.

### Divergence time

To estimate the divergence time between AKR and DKR, we used variation at four-fold degenerate sites using the high quality AKR (from AWC) and DKR (from AGA) samples, plus all of the kangaroo rat species with a completed genome available at NCBI (www.ncbi.nlm.nih.gov). Specifically, the assemblies of the Stephens’ kangaroo rat (SKR) (GCA_004024685.1), the Merriam’s kangaroo rat (MKR, *D. merriami*) (GCA_024711535.1), the Banner-tailed kangaroo rat (BKR, *D. spectabilis*) (GCF_019054845.1), the Ord’s kangaroo rat (OKR, *D. ordii*) (GCA_000151885.2), plus the outgroup genome of the pacific (little) pocket mouse (PPM, *Perognathus longimembris pacificus*) (GCA_004363475.1) were downloaded from NCBI. Single copy orthologs from the assemblies were retrieved using BUSCO employing the dataset mammalia_odb10. To get accurate codon-by-codon alignments, amino acid sequences were aligned using clustalw (Sievers et al. 2011) and used as reference for aligning nucleotide sequences using pal2nal (Suyama et al. 2006). The nucleotides at the four-fold degenerate sites were extracted and concatenated using a custom R script (Suppl. Material). These sites were used in the phylogenetic analysis.

To determine the phylogeny and divergence times, we used two approaches. First, we adopted a Bayesian approach implementing a strict molecular clock using Beast 2.7.7 (Bouckaert et al. 2019) with the Yule model selected for the tree prior. The Markov Chain Monte Carlo (MCMC) was run for 10 million states, and trees was sampled every 1000 states. The first 1000 (10%) trees were discarded as burn-in. The tree was visualized in FigTree v1.4.4 (Rambaut 2018). Second, we employed maximum likelihood without a clock constraint using PAUP (Swofford 2002). The tree was generated using heuristic search for random addition sequences and running 5 repetitions of TBR (tree bisection and reconnection) branch-swapping. The significance of the nodes was tested using 100 bootstraps. We also ran a maximum likelihood estimation with a clock using PAUP to compare to the Bayesian result.

To get the initial parameters for both programs, the best model and rates of nucleotide substitution were estimated by ModelTest-NG (Darriba et al. 2020), and Akaike Information Criterion (AIC) tests were used to compare the models. The trees were visualized using Evolview (Subramanian et al. 2019).

The branch lengths, scaled by the number of substitutions (*l*) were converted to divergence times given the assumed neutrality of four-fold degenerate sites. Neutral mutations fix at a rate equal to the neutral mutation rate (µ) per generation (Kimura 1968), so time since divergence (t) can be calculated using:

1 $${\rm{t}}=\mathrm{lg}/{\rm{\mu }}$$

The generation time (g) was set as 1.14 years, which was estimated for SKR using data from Price and Kelly (1994) (Suppl. Material), and the mutation rate (µ) was set as 5 × 10^−9^ per bp per generation, the middle of the range of 3.5–6.5 × 10^−9^ estimated for mice (Uchimura et al. 2015; Lindsay et al. 2019).

### Demographic history

The two samples with high quality sequencing, an AKR sample from AWC and a DKR sample from AGA, were used for genotype calling to get estimates of historical effective populations sizes (*N*_*e*_) of each taxon using a pairwise sequential Markovian coalescent (PSMC) model (Li and Durbin 2011). The PSMC method uses the heterogeneity in heterozygosity (i.e., SNPs) along the genome to define recombinant blocks. The coalescence time of the blocks is estimated by their divergence, and fewer regions coalescing at a certain time in the history implies a larger population size at that time. The model parameters were those used for *Rattus norvegicus* by Deinum et al. (2015), with the number of iterations (N) increased to 80 from the default 25 to promote the convergence of history inferences, the number of free parameters (p) was reduced from the default to prevent overfitting, and the initial mutation/recombination rate ratio *r* was set at 1.33 based on estimates from laboratory rats and mice (Jensen-Seaman et al. 2004; Deinum et al. 2015). The other parameters were set as default, giving settings of -N80 -t15 -r1.33 -b -p “2*4 + 18*2 + 4 + 6”. As in the calculation of divergence times, the estimation of *N*_*e*_ used a generation time (g) of 1.14 yrs and mutation rate (µ) of 5 × 10^−9^.

## Results

### Reference genome assembly and sequencing coverage

The individual from SCP (see Fig. 1) was used to prepare a reference genome. The PacBio assembly was 3.40 Gb, with 3569 contigs and a N50 of 10.88 M bp, with an average coverage of 23.59×. BUSCO retrieved 8778 (95.1%) of the genes in the database mammalia_odb10, including 160 (1.7%) duplicated genes and 95 (1.0%) fragmented genes, while 353 (3.9%) genes were missing.

The read coverage for the two samples with high quality sequencing was 20.27× for the AKR sample from AWC and 20.08× for the DKR sample from AGA that was sequenced using 10X linked reads. For all other samples, it averaged 5.24×.

### Species delineation

Genome-wide Fst values showed a clear distinction between the three northern populations (PHL, AWC, LTC) and the four southern populations (BXSH, BXSL, MRR, AGA) (see map, Fig. 1). They differed by an average Fst of 0.2086, while within these two groups Fst values were more than an order of magnitude lower, with an average of 0.0093 and 0.0032 respectively (Table 1). Since the average between-region Fst values (north vs. south) for each population were always much greater than the within-species comparison, a one-tailed permutation test shows the between-species values to be significantly greater (*p* = 3.40 × 10^−6^) without even taking account the magnitude of the difference. This pattern corresponded precisely to the expectation of AKR in these northern populations and DKR in the south (Fig. 1).

**Table 1:** The matrix of genome-wide Fst between AKR and DKR population pairs^a^ and the nucleotide diversity (π) of each population.

Fst	PHL	AWC	LTC	BXSH	BXSL	MRR	AGA
PHL	–						
AWC	0.00349	–					
LTC	0.00951	0.01482	–				
BXSH	0.20586	0.22199	0.20608	–			
BXSL	0.24676	0.26998	0.24476	0.00834	–		
MRR	0.20742	0.22352	0.20760	0.00739	0.01862	–	
AGA	0.15647	0.15501	0.15724	0.01053	−0.00423	−0.02147	–
Avg. π ±std	0.00125 ± 5.1E−05	0.00122 ± 6.8E−05	0.00128 ± 1.5E−04	0.00076 ± 4.2E−05	0.00080 ± 3.5E−05	0.00072 ± 5.5E−05	0.00074
	*n* = 10	*n* = 5	*n* = 7	*n* = 3	*n* = 7	*n* = 3	*n* = 1

AKR sites are underlined. Sample size = n.

^a^*PHL* Phelan, *AWC* Applewhite Camp, *LTC* Lytle Creek, *BXSH* Box Springs high, *BXSL* Box Springs low, *MRR* Motte Rimrock Reserve, *AGA* Aguanga.

**Fig. 2:**
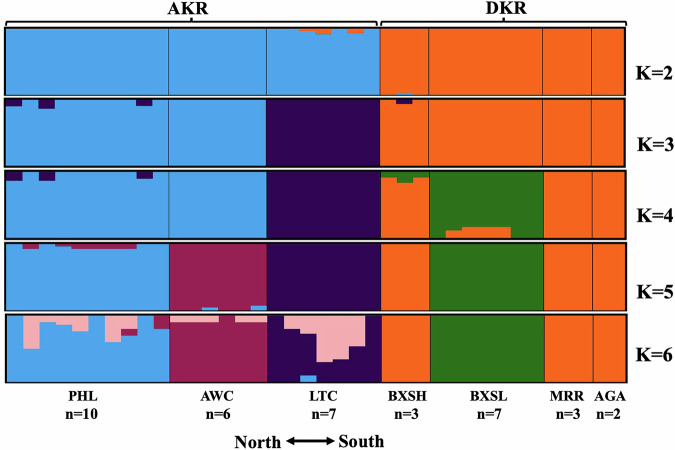
Genetic clusters defined by the genomes of individuals sampled along the North-South transect. *K* defines the number of clusters fitted by Admixture software, with *K* = 2 separating DKR and AKR. Sample sites are shown at the bottom, with the genetic composition of each of the *n* individuals sampled at a site defined by a vertical bar.

To detect signs of sympatry (as mixing within sites) or genetic introgression (as mixing within individuals) between the two species an Admixture analysis was performed. First, the analysis further confirmed the clear division of the individuals sampled into two groups (Fig. 2, K = 2). This division was the most strongly supported partitioning of the data, with Delta K = 1.15 × 10^10^ for K = 2 (vs. K = 1), while for all other transitions Delta K ranged from 0.26 to 1.42. A single very high value identifies the major partition (Evanno et al. 2005), in this case unambiguously supporting K = 2. Second, there was no evidence of sympatry, since no site included individuals of both groups, and as the number of partitions was increased there was no evidence of AKR/DKR hybridization. Even with K = 6, there was no indication of genetic introgression between the species, supporting their long-term genetic isolation (Fig. 2).

The PCA results showed genetic differentiation consistent with the Admixture analysis indicating the absence of any sympatry. All individuals from a given sample location clustered together, with the AKR and DKR samples clearly separated by PC1, which explained 30.1% of the variance (Supplementary Fig. S1).

These data (Fst, PCA, and Admixture) are consistent in providing strong support for the species-level status of AKR and DKR. The Admixture and PCA results show that within populations all individuals are either all AKR or DKR, and the Admixture analysis indicates that the two taxa are genetically distinct with no evidence of gene flow.

The clear genetic differentiation of AKR and DKR raises the question of what has maintained their separation. The genomic data showed that AKR is found across the San Gabriel Mountains, including northern (PHL), central (AWC), and southern (LTC) locations, showing that these mountains are not acting as a physical barrier separating AKR and DKR.

### Historical origins and effective population size

**Fig. 3:**
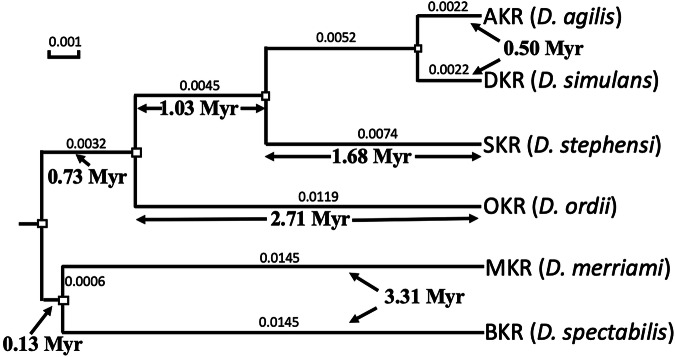
Bayesian phylogenetic tree of six kangaroo rat species (Genus *Dipodomys*) with a strict clock. The branch lengths are scaled by the rate of substitutions shown above the branches, and the estimated divergence times (using Eq. (1)) are shown below. The small boxes at the nodes represent 95% highest posterior density (HPD) intervals, and all the nodes have posterior probability of 1. PPM (not shown) was used as an outgroup to root the tree.

The Bayesian phylogenetic tree (chronogram) (Fig. 3) and the maximum likelihood phylogenetic tree (phylogram) (Supplementary Fig. S2) both show that DKR and AKR diverged roughly 0.3~0.6 mya, with the former dating the divergence to 0.50 (with a 95% highest posterior density of ±0.02) mya, and the latter estimating a mean divergence time of 0.46 mya (=mean of the two branches, with se of ±0.09). Both trees show that the DKR/AKR clade separated from SKR ~ 1.7 mya.

The two trees differ in the placement of MKR and BKR, the two kangaroo rat species most distantly related to AKR and DKR. MKR and BKR form a clade in the Bayesian chronogram (with 100% statistical support), a pattern replicated in the maximum likelihood tree that included a molecular clock (data not shown), while the BKR branch has a basal separation from the other kangaroo rat species in the phylogram (also with 100% statistical support). This difference did not affect the AKR/DKR analysis and may have resulted from a generation-time effect, since MKR is about 1/3 of the body weight of BKR and probably has a shorter generation time, while the other kangaroo rat species have similar intermediate body sizes (Reid 2006). The estimated generation time of BKR at 1.7 years (Swanson 2001) is markedly longer than the estimate for SKR of 1.14 years that we used for the scaling of time to generations, and would result in fewer neutral changes than expected.

**Fig. 4:**
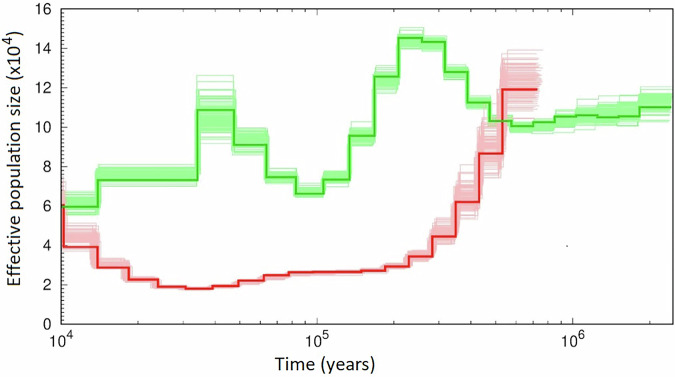
Historical effective population sizes of AKR and DKR. The green (AKR) and red (DKR) lines represent the estimated historical *N*_*e*_. The faint lines represent the 100-fold bootstrap results. The timescale assumes that both species have the same generation time.

Prior to the separation of AKR and DKR, the effective population size (*Ne*) of the ancestral taxon was estimated to be about 10^5^, and, at a time corresponding closely to the estimated divergence time, the *Ne* of DKR declined to around 2 × 10^4^ while the *Ne* of AKR stayed relatively constant around that pre-split level (Fig. 4). Around 35,000 years ago the effective size of AKR began to decline while that of DKR began to increase, both converging at *Ne* = ∼6 × 10^4^ some 10,000 years ago (Fig. 4). Importantly, since their separation, the effective population size (*Ne*) of each species has shown no indication of a severe bottleneck, noting that the decline in the DKR effective size after the split from AKR took several hundred thousand years to reach about 30,000, a level expected to have only a small effect in reducing genetic variation.

### Genomic polymorphism

The low coverage samples in both species showed nucleotide diversity estimates (π) ranging across sample sites from 0.00072 to 0.00080 (DKR) and 0.00122 to 0.00128 (AKR) (Table 1). The mean level of π across all individuals of the two species was about 64% greater in AKR than DKR (0.00126 vs. 0.00077; *t* = 17.91, df = 34, *p* < 2.2 × 10^−16^) (Supplementary Table S1).

The two species also differ significantly in an individual’s mean length of runs of homozygosity (ROHs). AKR individuals exhibited 56% longer ROH than DKR individuals (124,740 bp vs. 80,084 bp; *F* = 27.00, df = 1,29, *p* = 1.47 × 10^−5^), while within species there were no significant differences among populations (*F* = 1.520, df = 7,29, *p* = 0.200) (Supplementary Table S1). The longer ROHs in AKR probably indicate a shorter mean dispersal distance compared to DKR, resulting in greater ongoing local inbreeding in AKR due to a smaller neighborhood size. In this context, a neighborhood is the unit of spatial genetic structure that arises from isolation by distance within a continuously distributed population (Wright 1946). This difference in ROH length was not the result of a reference bias that could result if AKR had a smaller read depth (which could overestimate homozygosity) because the mean mapping read depth was greater in AKR than in DKR (5.55× vs. 4.75×, *t* = 3.394, df = 32.7, *p* = 0.0009).

## Discussion

Genomic data validated the decision of Sullivan and Best (1997) to divide PacKR into two distinct species, AKR and DKR, a distinction that Stock (1974) had previously considered possible based on their karyotype difference. However, we found no support for their suggestion, based on morphological measurements, that the species were sympatric over a large area south of the San Gabriel and San Bernadino Mountains: only a single species was found at each sampling location. Sullivan and Best (1997) noted that AKR is found at higher elevations, so we tested the hypothesis that within the proposed zone of sympatry AKR might be found at high elevation sites. In the Box Springs Mountains, a site within the proposed zone of sympatry (Fig. 1), we sampled a high and low elevation site in the (740 m vs 400 m); however, only DKR was found at both sites even though the high elevation site was higher than the AKR site of LTC (630 m) in the foothills of the San Gabriel Mountains.

The suggestion of extensive sympatry made by Sullivan and Best (1997) was based on a classification of individuals into the two species based primarily on skull morphology. Their method was developed from a comparison of individuals of the two taxa sampled more than 400 km apart and the validity of the species identification method was never tested using individuals of known karyotype from nearby populations. In contrast, Best et al. (1986) had previously found evidence of significant geographical variation in morphology within each taxon. Moreover, they had performed a morphological analysis using karyotyped individuals, finding that of their three 2 N = 62 (i.e. AKR) populations, the two high elevation populations (one each in the San Gabriel and San Bernardino Mountains) grouped together but the third, lower elevation, one (Devore, near LTC) grouped morphologically with two nearby 2 N = 60 (i.e. DKR) populations. These results further support the conclusion that the morphological separation used by Sullivan and Best (1997) to identify sympatry was unreliable. This morphological similarity aligns with the pattern seen very generally among kangaroo rat taxa; for example, even the phylogenetically well separated but sympatric DKR and SKR are very similar (see Chock et al. 2022, Table 1).

We found no evidence of historical or recent hybridization between the two species, which combined with the lack of evidence for sympatry, suggests their long-term genetic isolation from each other despite their small-scale geographical separation. Another pair of kangaroo rat sister species, the Panamint kangaroo rat (PKR) and Stephens’ kangaroo rat (SKR) are broadly sympatric with AKR and DKR, respectively. This clade separated from AKR and DKR about 1.7 mya (Fig. 3 & Supplementary Fig. S2), and it is believed that PKR and SKR separated soon after this as a result of the uplift of the San Gabriel and San Bernardino mountains, since neither species is found at higher elevations (Metcalf et al. 2001). In contrast, we found that AKR is found at higher elevations from north to south across the San Gabriel mountains, so that these mountains do not appear to be the geological barrier that created the AKR/DKR split. It can be seen from Fig. 1 that the Santa Ana River could have served and could still serve as a geological barrier separating AKR and DKR; however, the river flow is seasonal and has probably dried up during the late summer on many occasions, potentially reducing its effectiveness as a barrier. We did not sample from the north side of Santa Ana River, an area now heavily urbanized, so this hypothesis remains to be tested. However, as noted by Sullivan and Best (1997), a very plausible alternative hypothesis is that the marked habitat shift from relatively cool montane forest and upland chaparral to the hot and arid coastal sage scrub environment to the south of the mountains has maintained the isolation of these two taxa. This difference can be quantified in terms of the annual average rainfall at LTC (the lowest AKR site) vs. BXSH (the highest DKR site). The annual rainfall for LTC (elevation 630 m) is 92.7 cm while at BXSH (elevation 740 m) it is 39.4 cm (data for LTC from the Western Regional Climate Center; https://wrcc.dri.edu; for BXSH, from https://www.weatherworld.com/climate-averages). Thus, even at the highest elevation DKR site sampled (BXSH) the average annual rainfall is only 43% of the average at LTC, resulting in more arid-adapted vegetation.

A potentially similar example to the AKR/DKR case is provided by the Brazilian rodent, *Oecomys catherinae*, which shows a karyotype polymorphism in geographically separated populations in the Amazonian biome (2N = 62) and in the Atlantic Forest (2N = 60) (Malcher et al. 2017). Although the two forms exhibit very minor morphological and genetic (cytochrome b) differences, the authors proposed that they may represent two cryptic species resulting from chromosomal speciation. However, as in the AKR/DKR example, it is difficult to disentangle the effects of karyotype difference, allopatry and environmental factors in their divergence.

The estimated divergence time of AKR and DKR of roughly 0.5 mya is intermediate between two previously published estimates, both of which were based only on known geological/climatic events that could have plausibly been involved. Stock (1974) suggested that DKR and AKR (at that time still considered subspecies) were separated very recently, after the Wisconsin Pluvial (~13,000 yrs ago). In contrast, Sullivan and Best (1997) proposed a much earlier separation perhaps due to climate change causing marine transgressions or from the San Gorgonio Pass becoming a barrier about 2.5 mya during early-Pleistocene or perhaps even earlier. Under this scenario, they suggested that the ancestors of DKR were isolated in Baja California and later spread north to the Los Angeles Basin, becoming sympatric with AKR. Our genomic estimate does not support either, instead indicating a role of some other geological or climatic events in the mid- to late Pleistocene event causing their separation.

It is possible that our time estimate is biased downwards, since the generation time of 1.14 years may be an underestimate, bearing in mind that it is based on limited data from a different species, SKR. The only other kangaroo rat estimate available is 1.7 years for BKR (Swanson 2001); however, this animal is over twice the weight of AKR and DKR and found in a different area. On the other hand, SKR often co-occurs with DKR and so experiences similar environmental conditions. If we assume that the true value of the average generation time of these two species is somewhere between 1.14 and 1.7 years, then the divergence time separating AKR and DKR is in the range of 0.50–0.75 Myr.

The strict clock tree (Fig. 3) implicitly assumes that all species analyzed have the same generation time. This is not the case (comparing the SKR and the BKR estimates); however, the agreement of the estimates from this tree and the maximum likelihood tree average (which does not make the constant-generation-time assumption) is reassuring. The notable difference in the AKR and DKR branch lengths in the maximum likelihood tree (0.37 and 0.55 Myr; Supplementary Fig. S2) strongly suggests that these two species differ in their generation time. The shorter branch of the AKR lineage (indicating less evolutionary change per unit time) is consistent with a longer generation time that may be induced by the more northern, higher elevation habitat of this species.

The divergence time of ~0.5 mya for AKR and DKR is a relatively short period for sister species to develop; however, it is not inconsistent with the available estimates. The mean estimate for primate speciation is 1.1 mya (Curnoe et al. 2006), but more generally Avise et al. (1998) estimated that speciation of 50% of mammalian sister species occurred within the last 2 mya. In the present case, the genetic separation of AKR and DKR for about half a million years has resulted in them becoming significantly genetically differentiated (mean Fst = 0.2086 between species vs. 0.0052 within species) despite currently occurring within 25 km of each other (and possibly even closer). Thus the whole genome Fst between the two taxa is more than an order of magnitude greater than the differences between all population pairs within each taxon (Table 1). The very low within-taxon inter-population Fst persisted even when the populations sampled were separated by a substantial geographical distance (see Fig. 1), with the DKR populations of BKS and AGA being more than 60 km apart. Furthermore, the average Fst between AKR and DKR of 0.209 is large enough to be consistent with speciation. For example, it is greater than the difference between the Eurasian grey wolf and the coyote (Fst = 0.16; vonHoldt et al. 2016), two taxa that also show no evidence of hybridization.

A chromosomal rearrangement such as that separating AKR and DKR can be fixed in a population even if it has no advantage through the effects of genetic drift (given neutrality or weak underdominance). In particular, spatial structure enhances the chance of karyotype change, even within a large population, provided that dispersal is low enough for drift to fix a novel karyotype locally (Mackintosh et al. 2023). This local fixation can allow further spread to neighboring areas, particularly if local population density is subject to fluctuations. It is therefore notable that kangaroo rats typically have very limited dispersal. For example, the individual dispersal distance of SKR, a species commonly sympatric with DKR in the study area, is generally low (<40 m) (Price et al. 1994). This pattern suggests that habitat discontinuity may severely limit movement, which may be most apparent in the more mountainous habitat of AKR. The geographical distance separating the three AKR sample sites was much less than the distance between DKR sites (Fig. 1) and yet the genetic differentiation among sites was essentially identical in the two species (Table 1), with the most southern AKR site LTC showing a marked separation along PC2 (Supplementary Fig. S1). Supporting the view that dispersal is more limited in the mountains was the finding that the runs of homozygosity (ROH) were significantly longer in AKR. Long ROHs are long stretches of homozygous loci indicating genomic segments that are identical by descent from a recent common ancestor and as such are an indicator of the level of recent inbreeding rather than reflecting longer term effects such as the historical *N*_*e*_ (Ceballos et al. 2018; Brüniche-Olsen et al. 2018).

The finding of more population structure in AKR suggests that they may have been more prone to karyotype change than DKR, on the assumption that the habitat difference observed at the present time was a factor in the original divergence of the two taxa. In support of this view, it is parsimonious to conclude that the karyotype 2N = 60 found in DKR is ancestral, since *D. venustus* and *D. elephantinus*, close relatives of AKR and DKR (Alexander and Riddle 2005), both have 2N = 60. In contrast, Stock (1974) suggested that 2N = 62 was more probably ancestral because centric fusion was more common than fission in the intraspecific variants in kangaroo rats; however, this hypothesis requires 2N = 60 to have become fixed at least twice in the 4-species clade.

The levels of nucleotide diversity (π) in AKR (0.00126) and DKR (0. 00077) are comparable to the genome-wide diversity found in other rodent populations, such the house mouse (*Mus musculus domesticus*) in North America (0.0017; Ferris et al. 2021), brown rat (*Rattus norvegicus*) in China (0.0014; Chen et al. 2021), white-footed mouse (*Peromyscus leucopus*) in an outbred captive population (0.0033; Long et al. 2019), and cactus mouse (*Peromyscus eremicus*) in Southern California (0.0040; Motte population, estimated from Tigano et al. 2020). However, precise comparisons between genomic studies are complicated by differences in data filtering (Hemstrom et al. 2024).

The greater π in AKR populations compared to those of DKR is consistent with the larger historical *N*_*e*_ in AKR (Fig. 4). Although the longer ROHs in AKR indicate a smaller neighborhood size (*N*_*n*_) than DKR, reducing *N*_*n*_ generally has a minimal effect on the overall effective population size *N*_*e*_ (Nunney 2016). The smaller *Ne* of DKR, with a minimum of about 2 × 10^4^, over a period of more than 150,000 years, was small enough to expect some loss of variation due to the effects of drift, and even quite large populations can lose some genetic variance in quantitative characters (Lande 1995).

The historical effective size of the two taxa pre-split was estimated at about 100,000 (Fig. 4); however, just prior to the point of separation, about half a million years ago, the DKR estimate suggests a higher effective size than the AKR data. It is possible that this inconsistency is due to a generation time difference between the two species that was suggested by the maximum likelihood phylogeny noted earlier. Correcting for the possibility of a longer generation time in AKR stretches its curve to the right, and would allow the pre-split estimates to coincide.

The period of lowered *N*_*e*_ in DKR (Fig. 4) may have been the result of its geographic isolation and fragmentation in Baja California during late Pleistocene and early Holocene due to climatic fluctuation. The possibility is consistent with the isolated distribution of other small mammals with limited dispersal abilities in the Mexican fauna (Ceballos et al. 2010). Indeed, three taxa of kangaroo rats found in Baja California were originally classified as species (*D. peninsularis*, *D. paralius*, and *D. antiquarius*) but later identified as subspecies of DKR (Stock 1974; Best 1983), consistent with past subdivision of DKR.

The evidence suggests that historically neither species has experienced a severe reduction in population size (*N*_*e*_ always >20,000; Fig. 4), and there was no indication of a more recent bottleneck given their substantial population-level genetic diversity. This knowledge of their past effective size provides an important guideline for future monitoring and conservation of these species in ever-urbanizing Southern California where habitat has been rapidly disappearing. Notwithstanding the oft-cited 50/500 rule (Franklin 1980), an alternative guideline is to use historical estimates of *N*_*e*_ as a guide to the appropriate target for conservation (Nunney 2000), an approach more consistent with the challenges of rapid adaptation faced in a changing climate. For these two taxa, it is apparent that, in their previous history of persistence and adaptation, they have never been reduced to low numbers. Their numbers have always been substantially more than an order of magnitude greater than the *N*_*e*_ = 500 guideline, and data suggest that such a history is a good predictor of current success (Wilder et al. 2023); however, it also means that we have no indication of whether they will be able to thrive at lower numbers.

In summary, genomic analysis provided evidence of the long-term genetic separation of AKR and DKR, supporting the original division of these two taxa into two species by Sullivan and Best (1997) based primarily on their karyotype difference. Given the finding that AKR and DKR are genetically distinct, with no evidence of a history of genetic introgression, both taxa satisfy the criterion of the genetic species concept, where a species is a group of genetically compatible interbreeding natural populations that is genetically isolated from other such groups (Baker and Bradley 2006). There was also no evidence of the sympatry that had been inferred from small morphological differences in samples collected south of the mountains by Sullivan and Best (1997). Of course, this genomic evidence has no bearing on whether or not these two species have developed pre- or post-mating reproductive isolating mechanisms, only that there is no evidence of genetic mixing since the two taxa split. Similarly, the possibility of historical sympatry is not excluded (although the absence of any indication of genetic introgression makes this unlikely), but instead it is suggested that the ecological shift at the southern edge of the mountains probably maintains their separation despite the absence of a clear physical barrier preventing sympatry.

The ability to draw these conclusions shows how genomic data can overcome the problem of similar morphology in taxonomic studies, revealing, in this case, the absence of interspecific gene flow, differences in population structure and in historical *N*_*e*_. The rapidly increasing abundance of genomic data in non-model organisms can enrich our knowledge about their evolutionary history and help predict the trends of population changes under climate change.

## Supplementary information

Supplemental Table & Figures

4-fold site extraction & SKR Generation Time

Animals sampled and assembly statistics

## Data Availability

These sequence data have been submitted to GenBank (www.ncbi.nlm.nih.gov/genbank) under accession number (PRJNA1020972). Details of sample locations and sequencing are provided in the Supplementary Materials.
