# Supplementary material for: Genomic analysis of differentiation and demography of the formerly conspecific agile (Dipodomys agilis) and Dulzura (D. simulans) kangaroo rats
Source: Heredity (Edinb). 2025 Aug 25;134(9):519–28. doi: 10.1038/s41437-025-00789-3 (PMC12457632; doi:10.1038/s41437-025-00789-3)

**Supplemental Table & Figure Legends**

**Table S1.** Mean runs of homozygosity (ROHs) and genetic diversity (π) for all individuals

| Sample id | Species | Sample Site | Mean ROHs | π |
| --- | --- | --- | --- | --- |
| A10 | DKR | MRR | 68005.1806 | 0.000675061 |
| A11 | DKR | MRR | 50235.9066 | 0.00077777 |
| A12 | DKR | BXSL | 109027.633 | 0.000803492 |
| A3 | DKR | MRR | 79685.061 | 0.000693749 |
| B1 | DKR | BXSL | 55788.8608 | 0.000869295 |
| B2 | DKR | BXSL | 50366.1675 | 0.00079538 |
| B3 | DKR | BXSL | 71079.5665 | 0.000762493 |
| B4 | DKR | BXSL | 54619.8616 | 0.000789787 |
| B5 | DKR | BXSL | 63673.3935 | 0.00081868 |
| B6 | DKR | BXSL | 52459.9547 | 0.000774697 |
| B7 | DKR | BXSH | 84760.903 | 0.000810556 |
| B8 | DKR | BXSH | 141940.575 | 0.000728077 |
| B9 | DKR | BXSH | 77409.8795 | 0.000755967 |
| C3 | DKR | AGA | 76831.4665 | 0.000739812 |
| 5082-YC-19 | DKR | AGA | 165381.85 | high coverage |
|  |  | **Mean DKR** | **80084.41732** | **0.000771058** |
| C4 | AKR | LTC | 116450.142 | 0.001135145 |
| C5 | AKR | LTC | 59152.6676 | 0.001549946 |
| C6 | AKR | LTC | 130833.444 | 0.001148257 |
| C7 | AKR | LTC | 103438.659 | 0.00133865 |
| C8 | AKR | LTC | 97675.7894 | 0.001336126 |
| C9 | AKR | LTC | 160041.933 | 0.001181769 |
| C10 | AKR | LTC | 109177.843 | 0.001271146 |
| C11 | AKR | AWC | 16323.1727 | high coverage |
| C12 | AKR | AWC | 152047.33 | 0.001164626 |
| D1 | AKR | AWC | 143620.084 | 0.001138955 |
| D10 | AKR | PHL | 133836.707 | 0.001277523 |
| D11 | AKR | PHL | 174107.918 | 0.001257576 |
| D12 | AKR | PHL | 133612.606 | 0.001225857 |
| D2 | AKR | AWC | 153944.525 | 0.001244884 |
| D3 | AKR | AWC | 92731.9912 | 0.001296317 |
| D4 | AKR | AWC | 164789.32 | 0.001270906 |
| D5 | AKR | PHL | 110863.876 | 0.001209892 |
| D6 | AKR | PHL | 137977.141 | 0.001329024 |
| D7 | AKR | PHL | 159682.819 | 0.001299712 |
| D8 | AKR | PHL | 142468.197 | 0.001237305 |
| D9 | AKR | PHL | 145032.46 | 0.001314612 |
| E1 | AKR | PHL | 100648.231 | 0.001171191 |
| E2 | AKR | PHL | 130559.593 | 0.001217909 |
|  |  | **Mean AKR** | **124739.8456** | **0.001255333** |

**Figure S1. Principal component analysis (PCA) on genome-wide SNPs in samples from all sites.** The AKR samples (unfilled symbols) are separated from the DKR samples (filled symbols) by PC1. PC2 separates LTC (southern edge of the mountains) from the other two AKR populations.


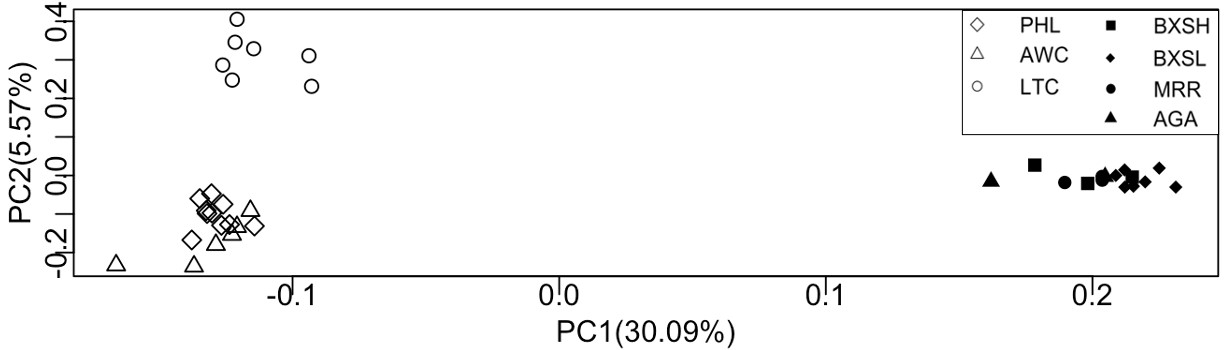


**Figure S2. Maximum likelihood phylogenetic tree of six kangaroo rat species (Genus *Dipodomys*).** No clock is imposed, so the branch lengths are scaled by the rate of substitutions shown above the branches, with the estimated divergence times (from equation 1) shown below, noting that this estimate assumes all species have the same generation time. All nodes have 100% bootstrap support. PPM (not shown) was used as an outgroup to root the tree.


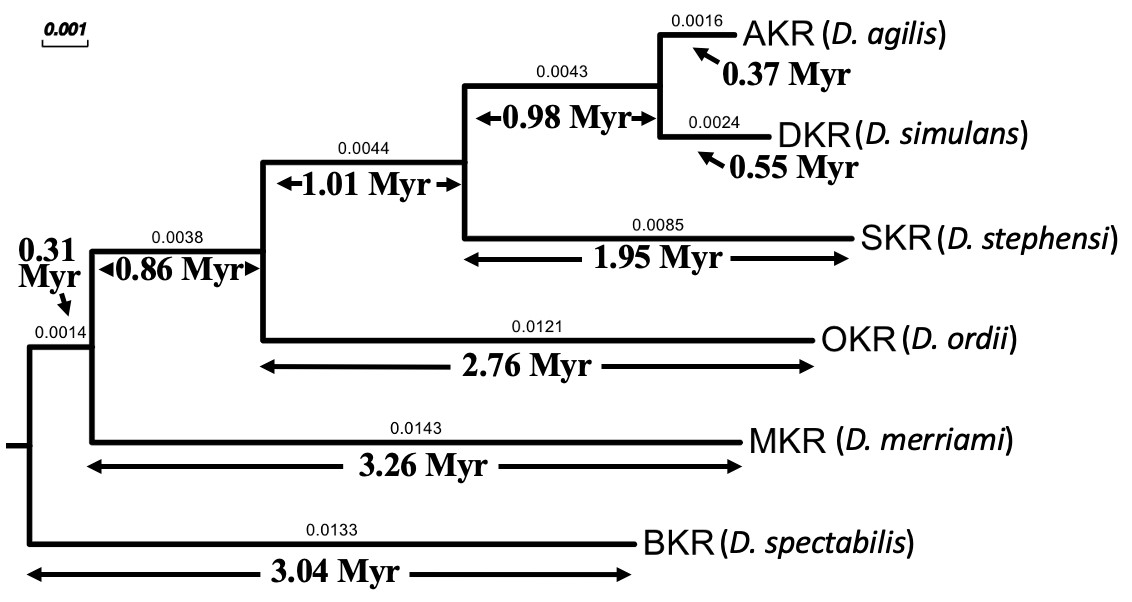

Supplement: Supplementary file 1 — Supplemental Table & Figures [file 41437_2025_789_MOESM1_ESM.docx]
